# When the past informs our future

**DOI:** 10.7554/eLife.67169

**Published:** 2021-03-09

**Authors:** Abigail LaBella

**Affiliations:** Department of Biological Sciences, Vanderbilt UniversityNashvilleUnited States

**Keywords:** placenta, endometrium, pregnancy, marsupial, eutheria, monotreme, Human, Other

## Abstract

Comparing the genes expressed at the maternal-fetal interface in different species helps to pinpoint those that contribute to a healthy pregnancy by regulating the activity of the immune system.

**Related research article** Marinić M, Mika K, Chigurupati S, Lynch VJ. 2021. Evolutionary transcriptomics implicates *HAND2* in the origins of implantation and regulation of gestation length. *eLife* **10**:e61257. doi: 10.7554/eLife.61257

Your belly button is the last reminder of arguably the most fascinating yet fleeting organ you ever had: the placenta. Simultaneously taking on the role of lungs, liver, gut and kidneys, this temporary structure develops during pregnancy to provide the fetus with oxygen and nutrients while also removing waste products (Burton and Fowden, 2015). The placenta also features the maternal-fetal interface, where the cells of the mother and the fetus work together to develop the structures and avenues of communication necessary for a successful pregnancy. Disrupting this process can result in complications such as preeclampsia or preterm birth, which affects around 15 million pregnancies worldwide every year (Blencowe et al., 2012; Erlebacher, 2013).

Yet, it is almost impossible to safely study the maternal-fetal interface during pregnancy: instead, researchers can turn to its evolutionary history. Indeed, how the interface came to be is inseparable from the way it operates and malfunctions today. In particular, comparing and contrasting this process across diverse species can highlight innovations in specific pathways or genes. Now, in eLife, Vincent Lynch and colleagues – including Mirna Marinić as first author, Katelyn Mika and Sravanthi Chigurupati – report on using this approach to identify the genes that the maternal-fetal interface needs to form and work properly in placental animals (Marinić et al., 2021).

Marinić et al. – who are based at the University of Buffalo, the University of Chicago and AbbVie – gathered gene expression data from the maternal side of the maternal-fetal interface from 28 species ranging from frogs to people. Of the 20,000 genes studied, just 149 had placental expression which, when mapped onto the evolutionary tree, suggested that these genes had started to be expressed at the maternal-fetal interface when placental structures first emerged.

One of the 149 genes, known as *HAND2* (short for heart and neural crest derivatives-expressed protein 2), encodes a regulatory protein that contributes to the embryo getting implanted into the wall of the uterus (Mestre-Citrinovitz et al., 2015). However, it has also been associated with preterm birth, and could be important later on for a healthy pregnancy (Brockway et al., 2019).

To understand how *HAND2* is involved in the later stages of pregnancy, Marinić et al. used short-tailed opossum data and human cell cultures to create a model for the role of this gene at the maternal-fetal interface. The experiments revealed that *HAND2* regulates *IL15*, a gene that encodes a cytokine – a type of small signaling protein that helps to regulate the activity of the immune system. While it was once thought that the maternal immune system was turned down to accommodate the ‘foreign cells’ of a fetus, scientists now know that it actively helps to establish and maintain a healthy pregnancy (Racicot et al., 2014). In particular, the immune system may contribute to triggering the onset of birth.

Marinić et al. discovered that early in pregnancy, *HAND2* is present at high levels at the maternal-fetal interface. In turn, this increases the local expression of *IL15*, which then recruits natural killer cells, a component of the immune system that shapes the maternal-fetal interface. As pregnancy progresses, the expression of *HAND2* and *IL15* decreases; this might aid in the correct timing of birth by reducing levels of natural killer cells at the interface (Figure 1C).

**Figure 1.**
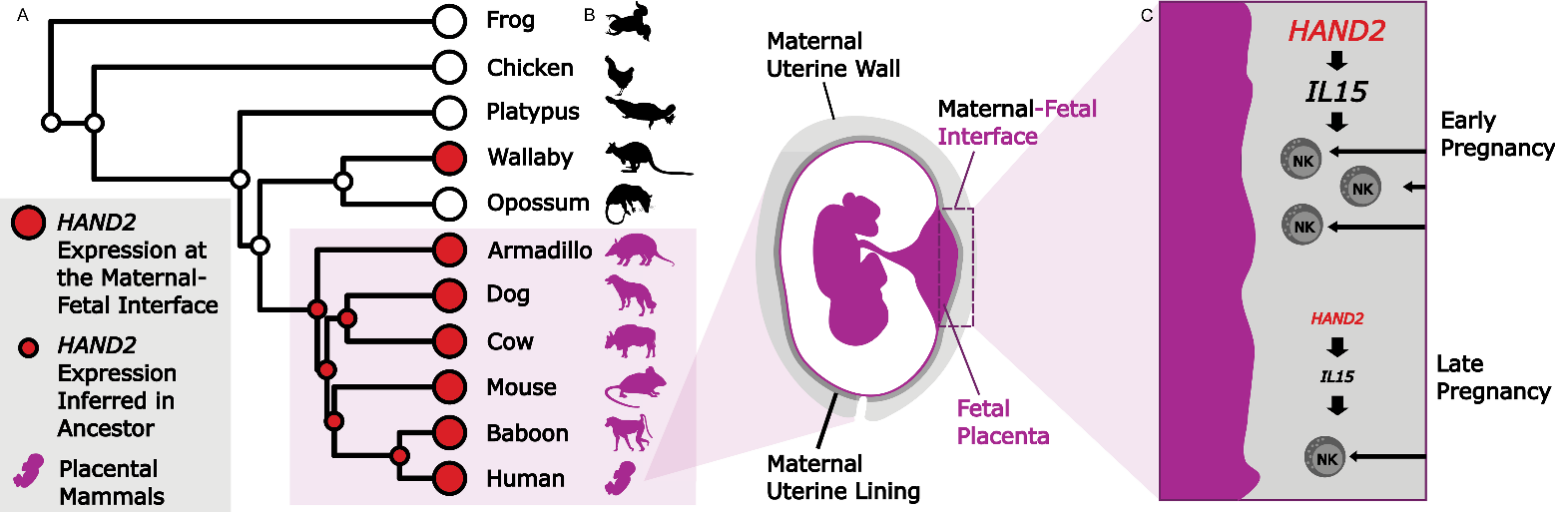
Studying evolution to understand the genes that shape the maternal-fetal interface. (**A**) The expression of *HAND2* (large red circles) was measured at the maternal-fetal interface across multiple placental and non-placental species. Marinić et al. then used ancestral reconstruction to infer that the expression of *HAND2* (small red circles) at the maternal-fetal interface arose in the ancestor of all placental mammals (shown in purple). In the wallaby, the expression of *HAND2* has a distinct evolutionary origin, which is most likely associated with other derived pregnancy traits in this species. (**B**) The placenta (fetal; purple) and uterine wall (maternal; gray) meet at the maternal-fetal interface, where nutrients and waste are exchanged, and communication systems are established. (**C**) During early pregnancy, *HAND2* is highly expressed at the maternal-fetal interface: the resulting increase in IL15 expression leads to immune natural killer cells (NK cells) being recruited to the interface (top), where they help to shape the structure. As pregnancy progresses, decreased *HAND2* and *IL15* expression alters the recruitment of NK cells, which may have a role in determining when birth should start.

To help diagnose, treat and prevent pregnancy disorders, we must first understand the tightly choreographed ballet of cells and communication signals that sustains healthy gestation. Examining the differences in gene expression between humans and closely related species helps to uncover new elements of this careful dance. By looking back at our past, Marinić et al. work to prevent future preterm births.
